# Ecological status and traditional knowledge of medicinal plants in Kedarnath Wildlife Sanctuary of Garhwal Himalaya, India

**DOI:** 10.1186/1746-4269-9-1

**Published:** 2013-01-02

**Authors:** Jahangeer A Bhat, Munesh Kumar, Rainer W Bussmann

**Affiliations:** 1 Department of Forestry and Natural Resources, H.N.B, Garhwal University, Srinagar-Garhwal, Uttarakhand, 249161, India; 2 William L. Brown Center, Missouri Botanical Garden, P.O. Box 299, St. Louis, MO, 63166-0299, USA

**Keywords:** Ethnomedicinal plants use, Ecological status, Resources, Altitudinal zone

## Abstract

**Background:**

Himalayan forests are the most important source of medicinal plants and with useful species for the local people. Kedarnath Wildlife Sanctuary (KWLS) is situated in the interior part of the Garhwal Himalayan region. The presented study was carried out in Madhmeshwar area of KWLS for the ecological status of medicinal plants and further focused on the ethnomedicinal uses of these plants in the study area.

**Methods:**

Ecological information about ethnomedicinal plants were collected using random quadrats in a random sampling technique along an altitudinal gradient in the KWLS. Information on medicinal properties of plants encountered in the present study was generated by questionnaire survey and was also compared with relevant literature.

**Results:**

A total of 152 medicinally important plant species were reported, in which 103 were found herbs, 32 shrubs and 17 were tree species which represented 123 genera of 61 families. A total of 18 plant species fell into the rare, endangered (critically endangered) and vulnerable status categories.

**Conclusion:**

The present study documented the traditional uses of medicinal plants, their ecological status and importance of these plants in the largest protected area of Garhwal Himalaya. This study can serve as baseline information on medicinal plants and could be helpful to further strengthen the conservation of this important resource.

## Introduction

The forests of India have been the source of traditional medicines for millennia. Of the 17,000 species of higher plants described in India, 7500 are known for their medicinal uses [1]. The Charak Samhita, a document on herbal therapy written about 300 BC, reports on the production of 340 herbal drugs and their indigenous uses [2]. The use of alternative medicine is growing because of its moderate costs and increasing faith in herbal medicine. Allopathic medicine can cure a wide range of diseases, however, its high prices and side-effects are causing many people to return to herbal medicines which tend to have fewer side effects [3]. A great amount of traditional knowledge about the use of medicinal plant species is still carried and orally transmitted by indigenous peoples. Regions with less accessibility and a comparatively slow rate of development, such as and mountainous areas like the Himalayas are excellent examples [4,5]. Because of the fast acceleration of market demand for herbal medicines, and recent controversies related to access, benefit sharing and bio-piracy, the documentation of indigenous knowledge is of urgent priority [6-10]. Indigenous knowledge, supplemented by the latest scientific insights, can offer new holistic models of sustainable development that are economically viable, environmentally benign and socially acceptable [11]. Currently, approximately 25% of allopathic drugs are derived from plant based compounds, and many others are synthetic analogues built on prototype compounds isolated from plant species [12]. According to the World Health Organization (WHO), as many as 80% of the world’s people depend on traditional medicine to meet their primary health care needs [13].

The Himalayan range in the northern part of India harbours a great diversity of medicinal plants. Of the approximately 8000 species of angiosperms, 44 species of gymnosperms and 600 species of pteridophytes that have been reported in the Indian Himalaya [14], 1748 species are known for their medicinal properties [15]. The state of Uttarakhand is a part of north-western Himalaya, and still maintains a dense vegetation cover (65%). The maximum species of medicinal plants have been reported from Uttarakhand [16,17], followed by Sikkim and North Bengal [15]. The trans-Himalaya in contrast sustains about 337 species of medicinal plants [4], which are low compared to other areas of the Himalaya due to the distinct geography and ecological marginal conditions [18]. Recent years have seen a sudden rise in the demand of herbal products and plant based drugs across the world resulting in the heavy exploitation of medicinal plants. Habitat degradation, unsustainable harvesting and over-exploitation to meet the demands of the mostly illegal trade in medicinal plants have already led to the extinction of more than 150 plant species in the wild [19]. More than 90% of plant species used in the herbal industries are extracted from the wild, and about 70% of the medicinal plants of Indian Himalaya are subject to destructive harvesting [20,21], and the majority of these plants stems from sub-alpine and alpine regions of the Himalaya [21]. The importance of ethnobiological knowledge on species-ecology can provide leads for new paths in scientific research and conservation, and has received growing attention in resource management worldwide [22,23]. International agencies such as the World Wildlife Fund (WWF) and United Nations Educational, Scientific and Cultural Orga-nization (UNESCO) as part of their people and plants initiative, are promoting research on ethnobotanical knowledge and the integration of people’s perceptions and practices in resource management at the local level [24].

The Kedarnath Wildlife Sanctuary (KWLS) is rich in biological diversity and is one of the most important regions of Garhwal Himalaya. The area of KWLS selected for this study is a particularly remote area, and the villagers residing in the area are fully dependent on forest resources, especially ethnomedicines for their daily livelihoods. Some ethnomedicinal studies on plants in this part of Himalayan region have been published, but hardly any ecological studies have also been carried out. The aim of the present study was to assess the ecological status of ethnomedicinal plants in a part of the largest protected area of Garhwal Himalaya.

## Materials and methods

### Study area

**Figure 1:**
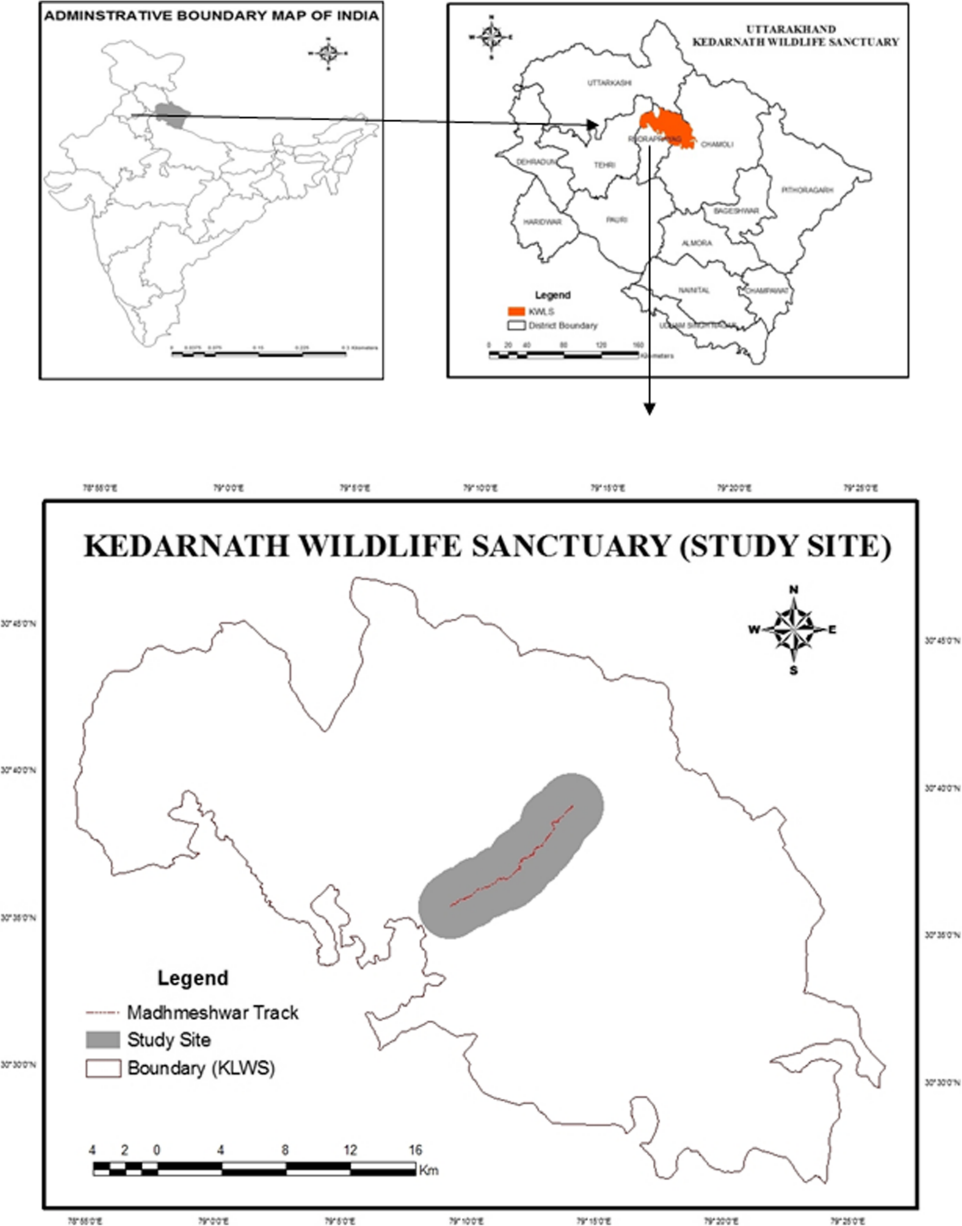
Map of the study area.

The present study was carried out in Madhmeshwar area, which is the interior part of Kedarnath Wildlife Sanctuary (KWLS) in the Western Himalaya of Chamoli-Rudraprayag districts of Uttarakhand, India. KWLS was established in 1972 and is situated in the north-eastern part of the Garhwal Himalayas between 30°25^′^-30°41^′^ N, 78°55^′^-79°22^′^ E. The Sanctuary falls under the IUCN management Category IV (Managed Nature Reserve) in the Biogeographical Province 2.38.12 of Himalayan highlands. KWLS is one of the largest protected areas with 97517.80 ha (25293.70 ha in Chamoli district and 72224.10 ha in Rudraprayag district) in the Western Himalaya [19]. The sanctuary lies in the upper catchment of the Alaknanda and Mandakini Rivers, which are major tributaries of Ganges. It is bordered by high mountain peaks, e.g. Kedarnath (6940 m), Mandani (6193 m) and Chaukhamba (7068 m) and harbors extensive alpine meadows, in particular Trijuginarayan, Kham, Mandani, Pandavshera, Manpai and Bansinarayan in the north, and several dense broad leaved oak mixed forest stands in the south. The present study represents data from a transect from the base of the mountain to the top in the Madhmaheshwer area between the coordinates 30°35^′^42^′^-30°38^′^12^′^N, 79°10^′^00^′^-79°13^′^00^′^E (Figure 1). The area receives 3000 mm of annual precipitation, of about 60% fall during the monsoon season (June-August). The relative humidity varies from 35 to 85% annually. There is moderate to heavy snowfall during December-February, even in low-altitude areas. The mean maximum temperature varies between 4°C (January) and 33.5°C (June).

### Ecological analysis of plant species

The vegetation analysis of ethnomedicinal plants was carried out following the stratified random sampling technique involving random quadrats. The size of the square plots was 100 m^2^ for trees, and nested within the main quadrats two plots of 25 m^2^ for shrubs and four plots of 1 m^2^ for herbs. The study area was divided into five altitudinal zones along the altitudinal gradient, to assess the ecological status of medicinal plant species. The frequency and density of all species was determined [25,26].

### Ethnomedicinal study of plant species

The plant species reported in the ecological studies of Madhmeshwar area were only taken for the ethnomedicinal study. A well structured questionnaire was prepared covering different questions regarding plants used for ethnomedicinal purposes. For the ethnomedicinal study only two villages were observed i.e., Gundhaar and Ransi in Madhmeshwar area and the respondents were selected randomly from the villages. Gundhaar is situated inside the sanctuary with 42 households while village Ransi is situated at the fringe of sanctuary with a total of 119 households. More than 10 percent of respondents of total population of the villages were selected for questionnaire survey. Both formal and informal discussions were carried out covering different age groups with both genders and mostly elders were involved in the interview process. The plants reported in ecological studies were also further used to collect the informations on ethnomedicinal uses with relevant available literature in Himalayan region and in a part of Kedarnath Wildlife Sanctuary [19,27,28]. The plant species having ethnomedicinal values were cross checked with the Red Data Book and other publications who have categorized the plant species under various threat (ecological status) categories [29-31].

### Collection and identification of plant specimens

From each sampling site, all plant species encountered in the quadrats were collected, and identified with the help of local and regional floras [27,32], while as some plants were also identified with the field guide [33]. Specimens collected during the surveys were processed in the laboratory according to [34]. These were pressed, dried in blotting sheets and poisoned with formaldehyde or mercuric chloride solution (0.5%) to protect against insect and fungal damage before mounting on the herbarium sheets. Voucher specimens were deposited in the Herbarium of Botanical Survey of India (BSD) and in the Herbarium of HNB Garhwal University Srinagar (GUH) under collector series JAB (Jahangeer Akbar Bhat). The nomenclature of the species follows “Flowering Plants of Uttarakhand - A Checklist” [35].

## Results and discussion

**Figure 2:**
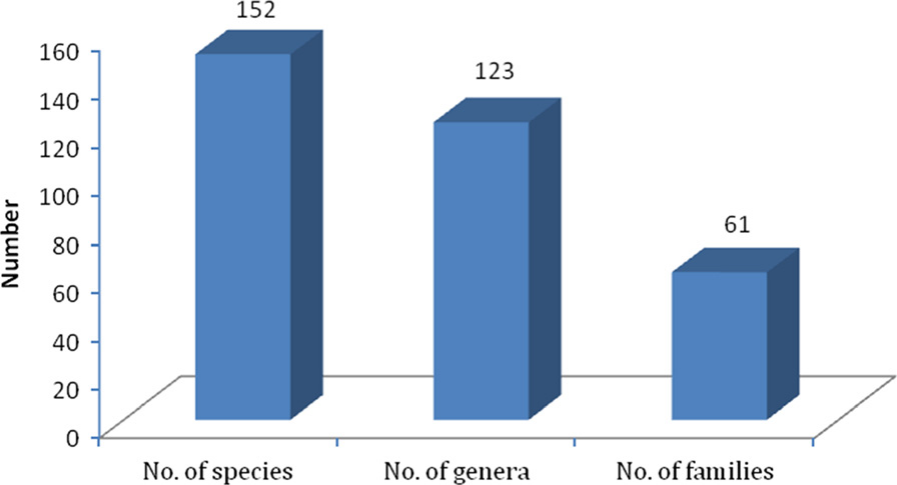
Total numbers of species, genera and families of plants having medicinal values.

**Figure 3:**
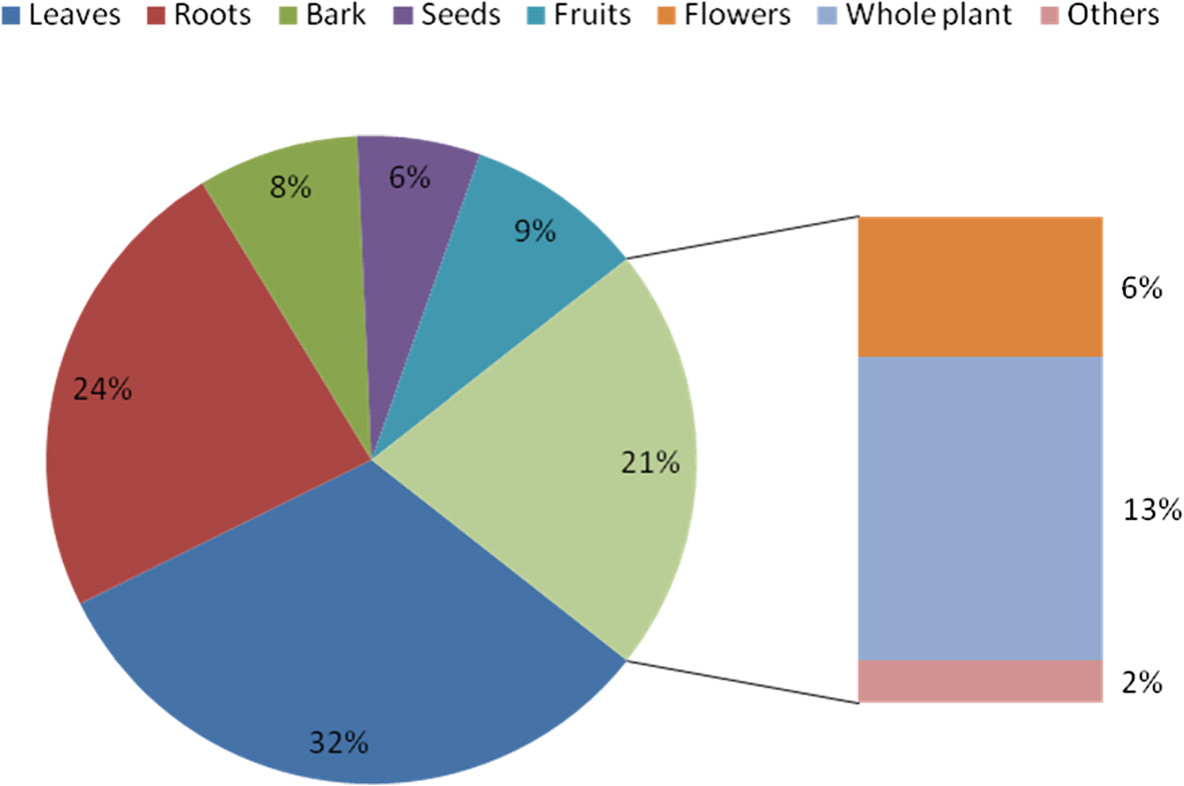
Percentage of plant parts used in preparing medicines for various ailments.

A total of one hundred and fifty two species of medicinally important plants (Table 1) were found in the quadrats including 49 species (Table 5) reported from the villages Gundhaar and Ransi of Madhmeshwar area. One hundred and three of these were herbs, thirty two shrubs and seventeen trees (Table 1), belonging to hundred twenty three genera of sixty one families (Figure 2). The most commonly used parts of ethomedicinal plants, compiled with relevant literature were leaves (32%), roots (24%), whole plants or plant (13%), followed by fruits (9%) and seeds and flowers (6% each) (Figure 3 and Table 2). According to different reports [19,27-31] eighteen plant species encountered have to be classified as rare, endangered, critically endangered or vulnerable (Table 2): *Aconitium hetrophyllum*, *Picrorhiza kurrooa*, *Podophyllum hexandrum*, *Rosa sericea*, *Roscoea alpina*, *Salvia hians*, *Saussurea auriculata*, *Sorbus aucuparia*, *Sorbus cuspidata*, *Synotis alatus*, *Bistorta amlexicaulis*, *Coriaria nepalensis*, *Hypericum choisianum* and *Morina longifolia* were recorded as rare species, while *Jurinea dolomiaea* and *Swertia chirayita* are classified as endangered, and *Polygonatum verticillatum* and *Zanthoxylum armatum* are vulnerable (Table 1).

**Table 1:** Plant species with their status and the part used in different ailments

**Scientific name**	**Accession No.**	**Status**	**Habit**	**Plant part used**	**Medicinal uses**
*Abies pindrow* Royle.	JAB-GUH-20578		T	Bark extract^2^	Cough & Bronchitis^2^
*Aconitium hetrophyllum* Wallich	JAB-BSD-114039	R^1^, Ce^3^, Vu^6^	H	Root^5^	Fever, cough,stomachache^5^
*Aesculus indica* (Wall. ex Cambess.) Hook.f.	JAB-GUH-20435		T	Seed paste^2^	Rheumatic Pain^2^
*Ainsliaea apetra* DC.	JAB-GUH-20677		H	Root extract^2^	Fever, painful urination^2^
*Ainsliaea latifolia* (D.Don) Sch.-Bip.	JAB-GUH-20680		H	Root decoction^2^	Colic^2^
*Anaphalis contorta* (D.Don) Hook.f.	JAB-GUH-20437		H	(Leaf & heads past, plant smoke)^2^	(Cuts, wounds & boils, insect repellent)^2^
*Anaphalis margaritaceae* (L.) Benth	JAB-GUH-20458		H	(Leaf & head paste)^2^	(Cuts, wounds & boils)^2^
*Anaphalis triplinervis* (Sims.) C.B. Clarke	JAB-GUH-20453		H	Leaf juice^2^, Flower^5^	Laceration of toes^2^, Dressing wounds^5^
*Anemone obtusiloba* D.Don	JAB-GUH-20619		H	Root decoction^2^	Diarrhoea^2^
*Anemone rivularis* Buch.-Ham. ex DC	JAB-GUH-20613		H	(Leaf past & juice)^2^ Leaves^5^	(Wounds^5^, sores & ear ache in local therapy)^2^
	JAB-BSD-114043				
*Arachne cordifolia* (Decne.) Hurusawa	JAB-GUH-20527		S	Leaf & Stem paste^2^	Wounds & Antidote to snake bite^2^
*Arisaema jacquemontii* Blume	JAB-GUH-20432		H	Fruits^2^, Tuber^5^	(Antidote of poisonous mushrooms & snake bite)^2^, (Cough, kidney &skin diseases)^5^
*Artemisia japonica* Thunb.	JAB-GUH-20446		H	(Leaves & flower tops)^2^	Incense & insecticide^2^
*Artemisia roxburghiana* Bess.	JAB-GUH-20468		H	Plant extract^2^	(Antipyretic, Tonic & also rubbed on skin allergy)^2^
*Asparagus filicinus* Buch.-Ham. ex D. Don	JAB-GUH-20436		H	Root tuberous^2^	(Diabetes, diarrhoea & dysentery)^2^
	JAB-BSD-114062				
*Aster peduncularis* Wallich	JAB-GUH-20687		H	(Plant extract & Root powder)^2^	(Renal-calculi & stomachic)^2^
*Barleria cristata* L.	JAB-GUH-20417		H	(Root decoction, Root & Leaves paste)^2^	(Bronchitis & pneumonia, wound swelling)^2^
*Begonia picta* Smith	JAB-GUH-20411		H	Plant decoction^2^	Colic & dyspepsia^2^
*Bergenia ciliata* (Haw.) Sternb.	JAB-GUH-20650		H	(Root^5^ rhizomatous)^2^	(Tonic, febrifuge, digestive & cutaneous disorders)^2^, (Fevers, diarrhoea & pulmonary infections)^5^
*Bidens bipinnata* L.	JAB-GUH-20440		H	Leaf juice^2^	(Leprosy initial stages, lactating mothers, cuts)^2^
*Bidens biternata* (Lour.) Merr. & Sherff	JAB-GUH-20441		H	Leaf juice^2^	(Leprosy initial stages, lactating mothers, cuts)^2^
*Bidens pilosa* L.	JAB-GUH-20444		H	(Plant extract & herbs of plants)^2^	(Cough & Bronchitis, leucoderma)^2^
*Bistorta amplexicaulis* (D.Don) Greene	JAB-GUH-20600		H	(Plant decoction & Leaf paste)^2^	(Cause abortion, wounds & relieves dysentery)^2^
*Bistorta vaccinifolia* (Wall. ex Meisn.) Greene.	JAB-BSD-114056	R^2^	H	Root decoction^2^	Tuberculosis^2^
*Blumea lanceolaria* (Roxb.) Druce	JAB-GUH-20679		H	Leaf paste^2^	Wounds & cuts^2^
*Buddleja asiatica* Lour.	JAB-GUH-20485		S	Leaf extract & Roots^2^	Skin diseases & Abortifacient^2^
*Bupleurum falcatum* L.	JAB-GUH-20427		H	Root decoction^2^	Fever & liver troubles^2^
*Calanthe tricarinata* Lindl.	JAB-GUH-20573		H	(Leaf paste Leaves & Pseudo-bulbs)^2^	(Sores & eczema, aphrodisiac)^2^
*Callicarpa arborea* Roxb.	JAB-GUH-20672		T	Bark^2^	Skin ailments^2^
*Cannabis sativa* L.	JAB-GUH-20488		H	Flowers^2^	Intoxicating agent^2^
*Carpinus viminea* Lindl.	JAB-GUH-20503		T	Leaves^5^	Bone fracture^5^
*Clematis buchananiana* DC.	JAB-GUH-20611		S	Leaf paste^2^	Skin ailments^2^
*Clematis montana* Buch.-Ham. ex DC.	JAB-GUH-20618		H	Leaf extract^2^	Diabetes & urinary troubles^2^
*Clinopodium umbrosum* (M.Bieb.) C. Koch	JAB-GUH-20558		H	(Plant extract & Leaf infusion)^2^	(Astringent, carminative, Blood purifier & Gastric troubles)^2^
*Corallodiscus lanuginosus* (Wall. ex DC.) B.L. Burtt	JAB-BSD-114064		H	Leaves^2^	Kidney stone^2^
*Coriaria nepalensis* Wallich	JAB-GUH-20502	R^2^	S	Fruits^2^	Emetic^2^
*Cotoneaster microphyllus* Wall. ex Lindl.	JAB-GUH-20640		S	Leaf, Fruits & Root Paste^2^	Diarrhoea, Cuts & Wounds^2^
*Cyathula capitata* Moq.	JAB-GUH-20422		H	(Leaf extract & Urticle)^2^	Emetic & abortifacient^2^
*Cyathula tomentosa* Moq.	JAB-GUH-20421		H	Leaf extract^2^	(Emetic property & given in snake bite)^2^
*Cynoglossum glochidiatum* Wall. ex Benth.	JAB-BSD-114059		H	Root extract^2^	Dyspepsia & digestive disorders^2^
*Cynoglossum lanceolatum* Forssk.	JAB-GUH-20481		H	Plant infusion^2^	Cold & cough^2^
*Debregeasia salicifolia* (D.Don) Rendle	JAB-GUH-20666		S	Bark^2^	Plaster for Bone Fracture^2^
*Delphinium vestitum* Wall. ex Royle	JAB-GUH-20616		H	Plant Stem^5^	Body swelling^5^
*Desmodium elagans* DC.	JAB-GUH-20531		S	Root infusion & Roots^2^	Epilepsy & Carminatives^2^
*Deutzia compacta* Craib.	JAB-GUH-20541		S	Leaves^2^	Diuretic^2^
*Dicliptera bupleuroides* Nees	JAB-GUH-20418		H	(Leaf^5^ paste & juice)^2^	(Wounds cough & gastro-enteritis)^2^ (Fever, skin diseases & stomachache)^5^
*Dipsacus inermis* Wallich	JAB-GUH-20483		H	Root paste^2^	Leucoderma & contusions^2^
*Elephantopus scaber* L.	JAB-GUH-20448		H	(Root extract & leaves)^2^	(Fever, stops vomiting, tonic for blood diseases)^2^
*Elsholtzia fruticosa* (D.Don) Rehder.	JAB-GUH-20551		S	Seeds^2^	Sciatica reliever^2^
*Elsholtzia strobilifera* Benth.	JAB-GUH-20549		H	(Plant^5^ paste)^2^	(Bruises & wounds^5^)^2^
*Eupatorium odenophorum* Spreng.	JAB-GUH-20452		S	Leaves^2,5^	Wounds^2^, Skin diseases^5^
*Euphorbia chamaesyce* L.	JAB-GUH-20410		H	Plant juice^2^	(Constipation & dysentery to infants)^2^
*Euphorbia hypericifolia* L.	JAB-GUH-20529		H	Leaf infusion^2^	(Dysentery, diarrhoea, menorrhagia)^2^
*Euphorbia pilosa* Linn.	JAB-GUH-20528		H	(Root decoction & Fruits)^2^, Seed & Leaves^5^	Constipation & emetic^2^, Food poisoning^5^
*Fagopyrum dibotrys* (D.Don) Hara	JAB-GUH-20597		H	Leaf paste^2^	Insect bite^2^
*Fragaria nubicola* Lindl. ex Lacaita	JAB-GUH-20628		H	Leaf juice^2^	Ear ache^2^
*Galinsoga parviflora* Cav.	JAB-GUH-20697		H	Plant extract^2^	Antidote of nettle sitting^2^
*Galium aparine* L.	JAB-GUH-20646		H	(Leaf extract & plant^5^ waste)^2^,	Astringent^5^, skin diseases^2^
*Galium asperifolium* Wallich.	JAB-GUH-20648		H	Plant waste^2^	Skin ailments^2^
*Geranium wallichianum* D. Don ex Sweet	JAB-BSD-114067		H	Root^5^ juice^2^	(Otorrhoea & opthalmia)^2^, (Dysentery & cold)^5^
*Gerbera gossypina* (Royle) P. Beauv.	JAB-GUH-20449		H	(Leaf juice & paste)^2^	(Cuts, wounds, plaster on bone fracture)^2^
	JAB-BSD-114060				
*Girardiana diversifolia* (Link) Friis	JAB-GUH-20670		H	Leaf juice^2^, Plant whole^5^	Gonorrhoea^2^, Diuretic^5^
*Gonatanthus pumilus* (D.Don) Engl. & Krause	JAB-GUH-20431		H	Root tuber paste^2^	Burns & wounds^2^
*Gonostegia hirta* (Blume) Miq	JAB-GUH-20669		H	Roots^2^	Plaster on fractured bones^2^
*Hippophae salicifolia* D.Don	JAB-GUH-20520		T	Fruits^2,5^	(Dandruff)^2^ & (Cardiac trouble)^5^
*Holmskioldia sanguinea* Retz.	JAB-GUH-20673		S	Leaf paste & Roots^2^	Body Swelling & Febrifuge^2^
*Hypericum choisianum* Wall. ex N. Robson	JAB-GUH-20691	R^2^	S	Leaf powder^2^	Fever^2^
*Impatiens scabrida* DC.	JAB-GUH-20474		H	Plant Stem^2^	Cause abortion^2^
*Indigofera heterantha* Wall. ex Brandis	JAB-GUH-20532		S	Leaf juice^2^	Diarrhoea, Dysentery & Cough^2^
*Inula cappa* (Buch.-Ham. ex D. Don) DC.	JAB-GUH-20456		S	Roots^2^	Suppressed urination^2^
*Juglans regia* L.	JAB- GUH-20520		T	Leaves^2^ , (Bark & Roots)^5^	Fungicide & Insecticide^2^ , Tooth ache^5^
*Jurinea dolomiaea* Boiss.	JAB-GUH-20443	E^3^	H	Root^5^	Incense, fever^5^
*Lamium album* L.	JAB-GUH-20559		H	Plant decoction^2^, Flower^5^	Contraceptive^2^, Bleeding after childbirth^5^
*Leptodermis Lanceolata* Wallich	JAB-GUH-20643		S	Bark paste^2^	Migraines^2^
*Leucas lanata* Benth.	JAB-GUH-20553		H	Plant infusion^2^	Whooping cough^2^
*Leycesteria formosa* Wallich	JAB-GUH-20494		S	Leaf paste^2^	Dandruff & Lice in hair^2^
*Lindenbergia indica* (L.) Vatke	JAB-GUH-20656		H	Leaves^2^	(Bronchitis, Cuts & wounds)^2^
*Lonicera angustifolia* Wall. ex DC.	JAB-GUH-20495		S	Fruits^2^	Gastric troubles of cattle^2^
*Lyonia ovalifolia* (Wallich) Drude	JAB-GUH-20524		T	Seed paste^2^	Wounds & Boils^2^
*Maianthemum purpureum* (Wall.) La Frankie	JAB-GUH-20565		H	Leaf extract^2^	Dysmenorrhoea^2^
*Morina longifolia* Wall. ex DC.	JAB-GUH-20571	R^2^	H	(Root^5^ paste & dried roots)^2^	(Wounds & incense)^2^, (Burns & boils)^5^
*Myrica esculenta* Buch.-Ham. ex D. Don	JAB-GUH-20702		T	Bark^2,4^ ,Leaves^5^ & Fruit^4,5^	(Intoxicate to fishes)^2^ (Vit. C, Asthama, Bronchitis, Diarrhoea & tooth ache)^4^ (Skin diseases & wounds)^5^
*Neolitsea pallens* (D.Don) Momiyama & Hara	JAB-GUH-20563		T	Fruits^2^	Scabies & Eczema^2^
*Nepeta ciliaris* Benth.	JAB-GUH-20552		H	(Leaf & seed decoction)^2^	Fever^2^
*Nomocharis oxypetala* (Royle.) E.H.Wilson.	JAB-GUH-20557		H	Bulb^5^	Vigorous^5^
*Origanum vulgare* L.	JAB-GUH-20561		H	Plant extract^2^, Leaves^5^	(Bronchitis, colic & diarrhoea)^2^, Toothache, swelling^5^
*Paeonia emodii* Wall. ex Royle	JAB-GUH-20575		H	(Roots & flower infusion)^2^, Tuber& leaves^5^	(Whooping cough, diarrhoea, intestinal spasms)^2^, Uterine diseases^5^
*Parnassia nubicola* Wall. ex Royle	JAB-GUH-20539		H	Root^5^ paste^2^	Antidote of snake bite^2^, Boils^5^
*Pedicularis hoffmeisteri* Klotz.	JAB-GUH-20657		H	Plant whole^5^	Food poisoning^5^
*Persicaria polystachya* (Wall. ex Meissn.) H. Gross	JAB-GUH-20598		S	Leaf paste^2^	Laceration of toes^2^
*Phalaris minor* Retz.	JAB-GUH-20591		H	Root paste^2^	Wounds^2^
*Picrorhiza kurrooa* Royle ex Benth.	JAB-GUH-20654	R^1^, CE^3^,Vu^6^	H	Root^5^	Fever, stomachache^5^
*Pimpinella acuminata* (Edgew.) C.B. Clarke	JAB-GUH-20428		H	Plant extract^2^	Diarrhoea & dysentery^2^
*Pimpinella diversifolia* DC.	JAB-GUH-20426		H	Plant extract^2^	(Digestive disorders, cold & cough)^2^
*Pinus roxburghii* Sargent	JAB-GUH-20701		T	Saw Dust^2^ & Aerial parts^4^	(Asthma & Bronchitis)^2^ , (Resin for cracked toes)^4^
*Plantago depressa* Willd.	JAB-GUH-20580		H	(Leaf & seed paste)^2^	(Cuts, wounds, piles)^2^
*Plantago himalaica* Pilger.	JAB-GUH-20579		H	Leaves^5^	Dysentery^5^
*Podophyllum hexandrum* Royle.	JAB-GUH-20592	R^1^,E^3,^ E^6^	H	Root^5^	Wounds^5^
*Polygonatum verticillatum* (L.) All.	JAB-GUH-20564	Vu^3^	H	(Root^5^ paste & powder)^2^	(Gastric problems5, wounds)^2^
*Primula denticulata* Sm.	JAB-GUH-20606		H	(Flower & root paste)^2^	(Diabetes & urinary ailments, lice killing)^2^
*Prinsepia utilis* Royle	JAB-GUH-20413		S	(Seed^5^ oil)^2^ & (Root^5^-bark)^2^	(Rheumatic pain, Diarrhoea)^2^ & (Pile, Stomach disorders)^5^
*Pyrus pashia* Buch.-Ham. ex D. Don	JAB- GUH-20699		T	Fruits^2,4,5^ & Bark^4^	(Digestive disorder)^2 ,5^(Astringent, Laxative, Anthelmintic, Febrifuge)^4^
*Ranunculus hirtellus* Royle.	JAB-GUH-20620		H	Plant paste^2^	Wounds^2^
*Reinwardtia indica* Dumort.	JAB-GUH-20566		H	Flowers^2^	Tongue wash^2^
*Rhamnus virgatus* Roxb.	JAB-GUH-20624		S	Bark paste & Fruits^2^	Eczema & Ring Worm, Emetic & Purgative^2^
*Rhododendron arboreum* Smith	JAB-GUH-20521		T	Flower^2,4^ , Bark^2^, & (Young Shoots)^5^	(Digestive and respiratory disorder)^2^ (tonic for heart, diarrhoea & dysentery)^4^ (Headache, Blood dysentery)^5^
*Rhus javanica* L.	JAB-GUH-20424		S	Fruits & Bark Paste^2^	Colic & Cholera, Swelling & Wounds^2^
*Rosa sericea* Lindl.	JAB-GUH-20626	R^2^	S	Flower juice^2^ & Fruits^5^	Bowel complaints^2^, (Headaches & Liver complaints)^5^
*Roscoea alpina* Royle	JAB-BSD-114063	R^2^	H	(Plant extract, leaf powder)^2^, Root^5^	(Tonic, cuts & wounds of cattle)^2^, (urinary diseases & tuberculosis)^5^
*Roylea cinerea* (D.Don) Baill.	JAB-GUH-20556		S	Leaves decoction^2^	Malarial fever^2^
*Rubia manjith* Roxb. ex Fleming	JAB-GUH-20647		S	(Roots^5^ & Flowers)^2^	(Tonic & Astringent, Bacillary Dysentery)^2^, (Lower blood pressure, Kidney stone)^5^
*Rubus nepalensis* (Hook.f.) Kuntze	JAB-GUH-20625		H	Root paste^2^	Burns & scalds^2^
*Rubus niveus* Thunb	JAB-GUH-20638		S	Fruit extract & Fruit juice^2^	Dysmenorrhoea & Antidote of snake bite^2^
*Rumex hastatus* D.Don	JAB-GUH-20603		H	Leaf extract^2^	(Cuts & wounds, nettle sitting reliever)^2^
*Rumex nepalensis* Spreng.	JAB-GUH-20602		H	Leaf^5^ infusion^2^	(Dysmenorrhoea, stomach ache)^2^, Etching^5^
*Salvia hians* Royle ex Benth.	JAB-GUH-20555	R^2^	H	Leaf juice^2^, Root^5^	(Arthritic, pain & eczema, body swelling)^2^, (cold, coughs & anxiety)^5^
*Salvia nubicola* Wall. ex Sw.	JAB-GUH-20560		H	(Leaf paste, Root^5^ extract)^2^	(Wounds, cold & cough)^2^, Fever^5^
*Sapindus mukorossi* Gaertn.	JAB-GUH-20649		T	Fruit^2,4^ & Seed^4^	Hair^2^, (Expectorant, antiepileptic, Emetic, febrifuge & Dental cares)^4^
*Sarcococca saligna* (D.Don) Muell.-Arg.	JAB-GUH-20486		S	Leaves^2^, Roots^5^	Joint pain^2^, Bawseer^5^
*Saussurea albescens* (DC.) Sch.-Bip.	JAB-GUH-20466		H	Flower heads^2^	Bronchitis reliever^2^
*Saussurea auriculata* (Spreng. ex DC.) Sch.-Bip.	JAB-BSD-114072	R^2^	H	Leaf paste^2^	Venereal diseases^2^
*Saxifraga diversifolia* Wall. ex Ser.	JAB-BSD-14071		H	Root extract^2^	Vermifuge^2^
*Selinum candollii* DC.	JAB-GUH-20409		H	Root^5^ powder^2^	(Asthma, cough, hysteria)^2^, Toothache^5^
*Senecio graciliflorus* DC.	JAB-GUH-20462		H	(Leaf paste & Juice of heads)^2^	(Ringworm diseases & insect bites, pussed ear)^2^
*Silene edgeworthii* Bocquet.	JAB-GUH-20499		H	(Leaf & young shoots juice)^2^	Eye infections^2^
*Solanum suratteuse* Burm.	JAB-GUH-20660		H	(Fruits & flower buds)^2^	(Fever, cough, asthama, gonorrhoea, eye ailments)^2^
*Solidago virgaurea* L.	JAB-BSD-114061		H	(Leaves & herb juice, Chewed roots)^2^	(Kidney troubles, asthma, rheumatism, wounds, throat irritation)^2^
*Sorbaria tomentosa* (Lindl.) Rehder	JAB-GUH-20637		S	Fruits (smoke)^2^	Asthama^2^
*Sorbus aucuparia* L.	JAB-GUH-20632	R^2^	T	Fruit extract^2^	Cough & Cold^2^
*Sorbus cuspidata* (Spach) Hedlund	JAB-GUH-20634	R^2^	T	Bark decoction^2^	Fever^2^
*Swertia chirayita* (Roxb. ex Fleming) Karsten	JAB-GUH-20538	E^3^, Vu^6^	H	Leaves^5^	Blood diseases^5^
*Swertia ciliata* (G.Don) Burtt.	JAB-BSD-114044		H	Plant extract^2^, Leaves^5^	Malaria^2^, Blood purifier^5^
*Synotis alatus* (Wall. ex DC.) C. Jeffrey & Chen.	JAB-GUH-20681	R^2^	H	Plant decoction^2^	Fever^2^
*Taraxacum officinale* Weber.	JAB-GUH-20465		H	Root^5^ extract^2^	(Migraines, hepatitis & head ache)^2^, Blood purifier^5^
*Taxus baccata* L.	JAB-GUH-20661		T	Bark^2,5^ & Bark Paste^2^	(Plaster on fractured bones Headache)^2^ & (Breast Pile)^5^
*Triumfetta rhomboidea* Jacq.	JAB-GUH-20662		H	(Root juice, Fruits & Leaves)^2^	(Cuts, delivery facilitation)^2^
*Urena lobata* L.	JAB-GUH-20568		H	Root paste^2^	(Body pain & rheumatism)2
*Urtica ardens* Link.	JAB-GUH-20668		H	(Seed oil & Leaf extract)^2^	(Sciatica, rheumatism, skin ailments, hair-wash for avoiding baldness)^2^
*Urtica dioica* L.	JAB-GUH-20664		H	(Seed oil & Leaf extract)^2^, Root^5^	(Sciatica, rheumatism, skin ailments, hair-wash for avoiding baldness)^2^, Boils^5^
*Valeriana hardwickii* Wallich	JAB-GUH-20671		H	(Root^5^ decoction & Root paste)^2^	(Urinary disorder, joint pains)^2^, Wounds^5^
*Verbascum thapsus* L.	JAB-GUH-20652		H	(Plant extract & Seeds)^2^, Leaf & flower^5^	(Asthma, bronchitis, narcotic)^2^, (Ulcers, tumors & piles)^5^
*Vernonia anthelmintica* (L.) Willd.	JAB-GUH-20455		H	Leaf powder^2^	(Intestinal disorder, fever & skin ailments)^2^
*Vernonia cinerea* (L.) Less.	JAB-GUH-20407		H	(Leaf extract & seeds)^2^	(Dysentery, cold & cough)^2^
*Veronica anagallis-aquatica* Linn.	JAB-GUH-20658		H	Plant juice^2^	(Cuts, burns & sores)^2^
*Viburnum cotinifolium* D.Don	JAB-GUH-20496		T	Bark decoction^2^	Hepatic & digestive disorder^2^
*Viburnum erubescens* Wall. ex DC.	JAB-GUH-20490		S	Leaves^2^	Insecticide^2^
*Viburnum grandiflorum* Wall ex DC.	JAB-GUH-20492		S	Bark Decoction^2^	Hepatic troubles^2^
*Viburnum nervosum* D.Don	JAB-GUH-20493		S	Bark Decoction^2^	Menorrhagia^2^
*Viola canescens* Wallich	JAB-GUH-20690		H	(Plant^4^ decoction, Root & Leaf^5^ juice)^2^	(Malarial fever, bronchitis, asthma, emetic, cuts & wounds)^2^, (Headache,cold, cough & malaria)^5^, (Expectorant, antipyretic, diaphoretic)^4^
*Woodfordia fruticosa* (L.) Kurz	JAB-GUH-20567		S	Leaves & bark, Dry flowers^2^	Febrifuge, Haemorrhoids^2^
*Zanthoxylum armatum* DC	JAB-GUH-20460	Vu^3^	S	Leaves & Fruits^2^ (Seed & Bark)^5^	Mouth wash^2^ & tooth ache^2,5^ (Infection in stored grain)^5^

**Abbreviation Habit**: T Tree, S = Shrub, H = Herb **Status:** R = Rare, Vu = Vulnerable, Ce = Critically Endangered, E = Endangered. **Superscript**: ^(1)^ = Red Data Book (IUCN, 1993), ^(2)^ = Gaur 1999, ^(3)^ = National Medicinal Plant Board (NMPB, 2003), ^(4)^ = Joshi et al. 2010, ^(5)^ = Singh & Rawat 2011, ^(6)^ = Semwal et al. 2007.) J.A.B = Jahangeer Akbar Bhat, GUH = Garhwal University Herbarium, BSD = Botanical Survey Dehradun.

**Table 2:** **Medicinal tree species in the study area (F- Frequency %, D- Density trees/100 m**^**2**^**)**

**Species**	**Family**	**Zone-I**		**Zone-II**		**Zone-III**		**Zone-IV**		**Zone-V**	
		**(1550-1750 m)**		**(2000-2200 m)**		**(2450-2650 m)**		**(2900-3100 m)**		**(3350-3550 m)**	
		**F**	**D**	**F**	**D**	**F**	**D**	**F**	**D**	**F**	**D**
*Abies pindrow*	Pinaceae	-	-	-	-	-	-	20	0.25	10	0.10
*Aesculus indica*	Hippocastanaceae	15	0.15	-	-	-	-	-	-	-	-
*Callicarpa arborea*	Verbenaceae	5	0.05	-	-	-	-	-	-	-	-
*Carpinus viminea*	Corylaceae	-	-	5	0.10	-	-	-	-	-	-
*Hippophae salicifolia*	Elaegnaceae	-	-	-	-	10	0.15	-	-	-	-
*Juglans regia*	Juglandaceae	5	0.15	5	0.05	-	-	-	-	-	-
*Lyonia ovalifolia*	Ericaceae	25	0.30	25	0.50	15	0.30	-	-	-	-
*Myrica esculenta*	Myricaceae	20	0.30	-	-	-	-	-	-	-	-
*Neolitsea pallens*	Lauraceae	30	0.45	5	0.05	-	-	-	-	-	-
*Pinus roxburghii*	Pinaceae	35	0.55	-	-	-	-	-	-	-	-
*Pyrus pashia*	Rosaceae	10	0.10	-	-	-	-	-	-	-	-
*Rhododendron arboreum*	Ericaceae	25	0.25	50	0.90	50	1.10	-	-	-	-
*Sapindus mukorossi*	Sapindaceae	10	0.15	-	-	-	-	-	-	-	-
*Sorbus aucuparia*	Rosaceae	-	-	-	-	-	-	-	-	25	0.25
*Sorbus cuspidata*	Rosaceae	-	-	-	-	15	0.15	-	-	-	-
*Taxus baccata*	Taxaceae	-	-	-	-	-	-	10	0.10	-	-
*Viburnum cotinifolium*	Caprifoliaceae	-	-	-	-	15	0.25	25	0.35	-	-

### Ecological study of plant species

#### Altitudinal zone-I (1550–1750 m)

In this altitudinal zone ten trees were reported having medicinal values. The highest density (0.55 trees/100 m^2^) and frequency (35%) was found for *Pinus roxburghii* followed by *Neolitsea pallens* (0.45 trees/100 m^2^ density with 30% frequency). The lowest density (0.05 trees/100 m^2^) and frequency (5%) was observed for *Callicarpa arborea* (Table 2). Sixteen medicinal shrub species were found in this altitudinal zone. The highest density and frequency (1.85 plants/25 m^2^ and 52.50% respectively) was recorded for *Debregeasia salicifolia* followed by *Woodfordia fruticosa* (0.58 plants/25 m^2^, frequency 17.50%). *Arachne cordifolia* and *Sarcococca saligna* were found with the lowest density and frequency (0.18 plants/25 m^2^, 7.50%) (Table 3). A total of twenty nine herb species with medicinal values were found. Among the herb species, the highest density (0.93 plants/m^2^) was observed for *Gonatanthus pumilus* followed by *Rumex hastatus* (0.69 plants/m^2^). The highest frequency was again reported for *Gonatanthus pumilus* (16.25%) followed by *Aster peduncularis* and *Cyathula tomentosa* (15%). The lowest density and frequency (0.05 plants/m^2^ and 1.25% respectively) was recorded for *Valeriana hardwickii* (Table 4).

**Table 3:** **Medicinal shrub species in the study area (F- Frequency %, D- Density plants/25 m**^**2**^**)**

**Species**	**Family**	**Zone-I**		**Zone-II**		**Zone-III**		**Zone-IV**		**Zone-V**	
		**(1550-1750 m)**		**(2000-2200 m)**		**(2450-2650 m)**		**(2900-3100 m)**		**(3350-3550 m)**	
		**F**	**D**	**F**	**D**	**F**	**D**	**F**	**D**	**F**	**D**
*Arachne cordifolia*	Euphorbiaceae	10.00	0.18	-	-	-	-	-	-	-	-
*Buddleja asiatica*.	Buddlejaceae	12.50	0.43	-	-	-	-	-	-	-	-
*Clematis buchananiana*	Ranunculaceae	-	-	-	-	5.00	0.20	-	-	-	-
*Coriaria nepalensis*	Coriariaceae	-	-	12.50	0.25	10.00	0.58	70.00	2.73	-	-
*Cotoneaster microphyllus*	Rosaceae	-	-	-	-	-	-	17.50	0.68	33.33	0.23
*Debregeasia salicifolia*	Urticaceae	52.50	1.85	7.50	0.35	-	-	-	-	-	-
*Desmodium elagans*	Fabaceae	-	-	7.50	0.20	5.00	0.18	-	-	-	-
*Deutzia compacta*	Hydrangeaceae	-	-	-	-	7.50	0.30	-	-	-	-
*Elsholtzia fruticosa*	Lamiaceae	-	-	10.00	0.25	12.50	0.25	-	-	-	-
*Eupatorium odenophorum*	Asteraceae	12.50	0.48	-	-	-	-	-	-	-	-
*Holmskioldia sanguinea*	Verbenaceae	10.00	0.38	-	-	-	-	-	-	-	-
*Hypericum choisianum*	Hyperiaceae	10.00	0.20	5.00	0.18	-	-	-	-	-	-
*Indigofera heterantha*	Fabaceae	12.50	0.35	15.00	0.68	40.00	1.53	-	-	-	-
*Inula cappa*	Asteraceae	-	-	-	-	12.50	0.40	-	-	-	-
*Leptodermis lanceolata*	Rubiaceae	15.00	0.43	-	-	-	-	-	-	-	-
*Leycesteria formosa*	Caprifoliaceae	-	-	-	-	5.00	0.15	7.50	0.38	-	-
*Lonicera angustifolia*	Caprifoliaceae	-	-	-	-	-	-	20.00	0.48	33.33	0.38
*Persicaria polystachya*	Polygonaceae	-	-	-	-	2.50	0.05	7.50	0.35	-	-
*Prinsepia utilis*	Rosaceae	10.00	0.25	-	-	-	-	-	-	-	-
*Rhamnus virgatus*	Rhamnaceae	15.00	0.33	-	-	-	-	-	-	-	-
*Rhus javanica*	Anacardiaceae	10.00	0.33	-	-	-	-	-	-	-	-
*Rosa sericea*	Rosaceae	-	-	-	-	12.50	0.65	40.00	1.63	26.53	1.23
*Roylea cinerea*	Lamiaceae	15.00	0.33	-	-	-	-	-	-	-	-
*Rubia manjith*	Rubiaceae	-	-	-	-	10.00	0.28	-	-	-	-
*Rubus niveus*	Rosaceae	10.00	0.28	17.50	0.85	35.00	1.45	-	-	-	-
*Sarcococca saligna*	Buxaceae	7.50	0.23	2.50	0.15	-	-	-	-	-	-
*Sorbaria tomentosa*	Rosaceae	-	-	-	-	12.50	0.28	-	-	-	-
*Viburnum erubescens*	Caprifoliacae	-	-	-	-	12.50	0.40	10.00	0.75	-	-
*Viburnum grandiflorum*	Caprifoliacae	-	-	-	-	15.00	0.43	32.50	1.05	-	-
*Viburnum nervosum*	Caprifoliacae	-	-	-	-	-	-	12.50	0.38	-	-
*Woodfordia fruticosa*	Lythraceae	17.50	0.58	-	-	-	-	-	-	-	-
*Zanthoxylum armatum*	Rutaceae	10.00	0.33	-	-	-	-	-	-	-	-

**Table 4:** **Medicinal herb species in the study area (F- Frequency %, D- Density plants/m**^**2**^**)**

**Species**	**Family**	**Zone-I**		**Zone-II**		**Zone-III**		**Zone-IV**		**Zone-V**	
		**(1550-1750 m)**		**(2000-2200 m)**		**(2450-2650 m)**		**(2900-3100 m)**		**(3350-3550 m)**	
		**F**	**D**	**F**	**D**	**F**	**D**	**F**	**D**	**F**	**D**
*Aconitium hetrophyllum*	Ranunculaceae	-	-	-	-	-	-	-	-	18.75	0.33
*Ainsliaea apetra*	Asteraceae	-	-	-	-	-	-	31.25	0.95	-	-
*Ainsliaea latifolia*	Asteraceae	-	-	32.50	1.30	11.25	0.16	-	-	-	-
*Anaphalis contorta*	Asteraceae	-	-	-	-	10.00	0.20	23.75	0.69	-	-
*Anaphalis margaritaceae*	Asteraceae	-	-	-	-	6.25	0.16	10.00	0.16	-	-
*Anaphalis triplinervis*	Asteraceae	6.25	0.21	8.75	0.20	-	-	-	-	-	-
*Anemone obtusiloba*	Ranunculaceae	-	-	-	-	-	-	-	-	15.00	0.26
*Anemone rivularis*	Ranunculaceae	-	-	3.75	0.10	11.25	0.26	-	-	-	-
*Arisaema jacquemontii*	Araceae	-	-	-	-	8.75	0.23	11.25	0.29	-	-
*Artemisia japonica*	Asteraceae	-	-	13.75	0.34	-	-	-	-	-	-
*Artemisia roxburghiana*	Asteraceae	-	-	-	-	7.50	0.26	2.50	0.05	-	-
*Asparagus filicinus*	Asparagaceae	-	-	3.75	0.05	10.00	0.33	-	-	-	-
*Aster peduncularis*	Asteraceae	15.00	0.30	-	-	-	-	-	-	-	-
*Barleria cristata*	Acanthaceae	10.00	0.25	-	-	-	-	-	-	-	-
*Begonia picta*	Begoniaceae	-	-	-	-	-	-	8.75	0.21	-	-
*Bergenia ciliate*	Saxifragaceae	-	-	3.75	0.08	10.00	0.18	3.75	0.09	-	-
*Bidens bipinnata*	Asteraceae	-	-	6.25	0.15	-	-	-	-	-	-
*Bidens biternata*	Asteraceae	5.00	0.20	-	-	-	-	-	-	-	-
*Bidens pilosa*	Asteraceae	-	-	12.50	0.24	-	-	-	-	-	-
*Bistorta amplexicaulis*	Polygonaceae	-	-	-	-	10.00	0.36	21.25	0.61	2.50	0.21
*Bistorta vaccinifolia*	Polygonaceae	-	-	-	-	-	-	-	-	7.50	0.13
*Blumea lanceolaria*	Asteraceae	7.50	0.20	-	-	-	-	-	-	-	-
*Bupleurum falcatum*	Apiaceae	-	-	8.75	0.18	-	-	-	-	-	-
*Calanthe tricarinata*	Orchidaceae	-	-	-	-	-	-	-	-	11.25	0.20
*Cannabis sativa*	Cannabinaceae	-	-	6.25	0.25	-	-	-	-	-	-
*Clematis montana*	Ranunculaceae	11.25	0.33	-	-	-	-	-	-	-	-
*Clinopodium umbrosum*	Lamiaceae	3.75	0.09	8.75	0.16	-	-	-	-	-	-
*Corallodiscus lanuginosus*	Gesneriaceae	-	-	-	-	12.50	0.16	-	-	-	-
*Cyathula capitata*	Amaranthaceae	2.50	0.06	7.50	0.24	-	-	-	-	-	-
*Cyathula tomentosa*	Amaranthaceae	15.00	0.59	-	-	-	-	-	-	-	-
*Cynoglossum glochidiatum*	Boraginaceae	-	-	8.75	0.21	-	-	-	-	-	-
*Cynoglossum lanceolatum*	Boraginaceae	8.75	0.26	-	-	-	-	-	-	-	-
*Delphinium vestitum*	Ranunculaceae	-	-	-	-	-	-	-	-	8.75	0.16
*Dicliptera bupleuroides*	Acanthaceae	-	-	12.50	0.20	-	-	-	-	-	-
*Dipsacus inermis*	Dipsacaceae	-	-	-	-	5.00	0.09	-	-	-	-
*Elephantopus scaber*	Asteraceae	7.50	0.13	-	-	-	-	-	-	-	-
*Elsholtzia strobilifera*	Lamiaceae	-	-	-	-	-	-	10.00	0.25	3.75	0.10
*Euphorbia chamaesyce*	Euphorbiaceae	8.75	0.14	-	-	-	-	-	-	-	-
*Euphorbia hypericifolia*	Euphorbiaceae	-	-	-	-	-	-	-	-	6.25	0.16
*Euphorbia pilosa*	Euphorbiaceae	3.75	0.09	-	-	8.75	0.25	12.50	0.38	-	-
*Fagopyrum dibotrys*	Polygonaceae	-	-	7.50	0.23	7.50	0.14	-	-	-	-
*Fragaria nubicola*	Rosaceae	12.50	0.13	7.50	0.18	10.00	0.20	-	-	-	-
*Galinsoga parviflora*	Asteraceae	-	-	-	-	8.75	0.18	-	-	-	-
*Galium aparine*	Rubiaceae	-	-	-	-	-	-	8.75	0.20	6.25	0.13
*Galium asperifolium*	Rubiaceae	-	-	-	-	-	-	-	-	3.75	0.10
*Geranium wallichianum*	Gerianiaceae	-	-	-	-	8.75	0.26	5.00	0.15	-	-
*Gerbera gossypina*	Asteraceae	11.25	0.21	-	-	-	-	-	-	-	-
*Girardiana diversifolia*	Urticaceae	12.50	0.61	6.25	0.25	-	-	-	-	-	-
*Gonatanthus pumilus*	Araceae	16.25	0.93	-	-	-	-	-	-	-	-
*Gonostegia hirta*	Urticaceae	2.50	0.08	-	-	-	-	-		-	-
*Impatiens scabrida*	Balsamaniceae	-	-	-	-	-	-	3.75	0.09	-	-
*Jurinea dolomiaea*	Asteraceae	-	-	-	-	-	-	-	-	5.00	0.08
*Lamium album*	Lamiaceae	-	-	6.25	0.11	-	-	-	-	-	-
*Leucas lanata*	Lamiaceae	-	-	-	-	7.50	0.18	-	-	-	-
*Lindenbergia indica*	Scrophulariaceae	-	-	-	-	12.50	0.24	-	-	-	-
*Maianthemum purpureum*	Liliaceae	-	-	-	-	-	-	-	-	3.75	0.05
*Morina longifolia*	Morinaceae	-	-	3.75	0.13	3.75	0.15	6.25	0.11	-	-
*Nepeta ciliaris*	Lamiaceae	-	-	11.25	0.16	-	-	-	-	-	-
*Nomocharis oxypetala*	Liliaceae	-	-	-	-	-	-	-	-	11.25	0.13
*Origanum vulgare*	Lamiaceae	-	-	-	-	-	-	-	-	3.75	0.09
*Paeonia emodii*	Paenoniaceae	5.00	0.18	1.25	0.05	-	-	-	-	-	-
*Parnassia nubicola*	Saxifragaceae	-	-	-	-	-	-	15.00	0.21	-	-
*Pedicularis hoffmeisteri*	Scrophulariaceae	-	-	-	-	-	-	-	-	12.50	0.21
*Phalaris minor*	Poaceae	6.25	0.33	-	-	-	-	-	-	-	-
*Picrorrhiza kurrooa*	Scrophulariaceae	-	-	-	-	-	-	-	-	12.50	0.23
*Pimpinella acuminata*	Apiaceae	-	-	12.50	0.15	5.00	0.06	-	-	-	-
*Pimpinella diversifolia*	Apiaceae	-	-	7.50	0.13	-	-	-	-	8.75	0.19
*Plantago depressa*	Plantaginaceae	-	-	-	-	-	-	-	-	10.00	0.16
*Plantago himalaica*	Plantaginaceae	-	-	-	-	-	-	-	-	10.00	0.20
*Podophyllum hexandrum*	Podophyllaceae	-	-	-	-	-	-	-	-	11.25	0.19
*Polygonatum verticillatum*	Liliaceae	-	-	-	-	8.75	0.11	-	-	-	-
*Primula denticulate*	Primulaceae	-	-	-	-	-	-	-	-	18.75	0.31
*Ranunculus hirtellus*	Ranunculaceae	-	-	-	-	-	-	-	-	20.00	0.38
*Reinwardtia indica*	Linaceae	-	-	5.00	0.08	7.50	0.10	-	-	-	-
*Roscoea alpine*	Zingiberaceae	-	-	-	-	-	-	13.75	0.19	-	-
*Rubus nepalensis*	Rosaceae	-	-	-	-	-	-	38.75	1.15	5.00	0.11
*Rumex hastatus*	Polygonaceae	12.50	0.69	-	-	-	-	-	-	-	-
*Rumex nepalensis*	Polygonaceae	-	-	26.25	1.13	23.75	0.66	-	-	27.50	0.71
*Salvia hians*	Lamiaceae	-	-	-	-	-	-	-	-	12.50	0.21
*Salvia nubicola*	Lamiaceae	-	-	-	-	-	-	-	-	3.75	0.06
*Saussurea albescens*	Asteraceae	-	-	6.25	0.09	16.25	0.71	-	-	-	-
*Saussurea auriculata*	Asteraceae	-	-	-	-	-	-	-	-	13.75	0.19
*Saxifraga diversifolia*	Saxifragaceae	-	-	-	-	-	-	10.00	0.15	-	-
*Selinum candollii*	Apiaceae	-	-	-	-	-	-	-	-	8.75	0.20
*Senecio graciliflorus*	Asteraceae	-	-	-	-	-	-	-	-	8.75	0.15
*Silene edgeworthii*	Caryophyllaceae	-	-	-	-	12.50	0.23	7.50	0.14	-	-
*Solanum suratteuse*	Solanaceae	8.75	0.16	-	-	-	-	-	-	-	-
*Solidago virgaurea*	Asteraceae	-	-	-	-	8.75	0.13	-	-	-	-
*Swertia chirayita*	Gentianaceae	-	-	-	-	-	-	12.50	0.21	-	-
*Swertia ciliate*	Gentianaceae	-	-	-	-	-	-	-	-	18.75	0.30
*Synotis alatus*	Asteraceae	-	-	-	-	7.50	0.13	-	-	-	-
*Taraxacum officinale*	Asteraceae	-	-	-	-	-	-	-	-	7.50	0.13
*Triumfetta rhomboidea*	Tiliaceae	8.75	0.21	-	-	-	-	-	-	-	-
*Urena lobata*	Malvaceae	6.25	0.15	-	-	-	-	-	-	-	-
*Urtica ardens*	Urticaceae	2.50	0.20	-	-	-	-	-	-	-	-
*Urtica dioica*	Urticaceae	7.50	0.49	6.25	0.25	-	-	-	-	-	-
*Valeriana hardwickii*	Valerianaceae	1.25	0.05	-	-	-	-	-	-	-	-
*Verbascum thapsus*	Scrophulariaceae	2.50	0.10	3.75	0.05	5.00	0.08	11.25	0.30	-	-
*Vernonia anthelmintica*	Asteraceae	-	-	-	-	7.50	0.16	-	-	-	-
*Vernonia cinerea*	Asteraceae	-	-	7.50	0.15	-	-	-	-	-	-
*Veronica anagallis-aquatica*	Scrophulariaceae	2.50	0.10	-	-	18.75	0.61	-	-	-	-
*Viola canescens*	Violaceae	-	-	-	-	-	-	10.00	0.19	-	-

### ***Altitudinal zone-II (2000–2200 m)***

In this altitudinal zone-II, five trees, eight shrubs and twenty nine herbs with medicinal values were observed (Table 2, Table 3 and Table 4 respectively). The highest density (0.90 trees/100 m^2^) and frequency (50%) was found for *Rhododendron arboreum* followed by *Lyonia ovalifolia* (0.50 trees/100 m^2^, 25%). The lowest density (0.05 trees/100 m^2^) and frequency (5%) was observed for both *Juglans regia* and *Neolitsea pallens* (Table 2). The highest density and frequency for shrubs (0.85 plants/25 m^2^, 17.50%) was recorded for *Rubus niveus*, followed by *Indigofera heterantha* (0.68 plants/25 m^2^, 15%) values. The lowest density (0.15 plants/25 m^2^, 2.50%) was reported for *Sarcococca saligna* (Table 3). A total of twenty nine herbs were found with medicinal values and the highest density (1.30 plants/m^2^) and frequency (32.50%) was observed for *Ainsliaea latifolia*, followed by *Rumex nepalensis* (1.13 plants/m^2^, 26.25%). The lowest density (0.05 plants/m^2^) was reported for *Asparagus filicinus*, *Paeonia emodii*, *Verbascum thapsus*, *Bergenia ciliata*, and *Reinwardtia indica* (0.08 plants/m^2)^. The lowest frequency (1.25%) was recorded for *Paeonia emodii* (Table 4).

#### Altitudinal zone-III (2450–2650 m)

In this altitudinal zone-III, five trees with medicinal values were reported. Among these medicinal tree species, the highest density (1.10 trees/100 m^2^) and frequency (50%) was observed for *Rhododendron arboreum*. The lowest density (0.15 trees/100 m^2^) was recorded for *Hippophae salicifolia* and *Sorbus cuspidata* while as lowest frequency (10%) was observed for *Hippophae salicifolia* (Table 2). Fifteen shrub species with medicinal values were found in this altitudinal zone. The highest density and frequency (1.53 plants/25 m^2^ and 40%) was recorded for *Indigofera heterantha* followed by *Rubus niveus* (1.45 plants/25 m^2^, 35%), while the lowest density and frequency (0.05 plants/25 m^2^, 2.50%) was registered for *Persicaria polystachya* (Table 3). In the herb layer thirty (30) species were found. Among these *Rumex nepalensis* had the highest density and frequency (0.66 plants/m^2^, 23.75%), followed by *Veronica anagallis*-*aquatica* (0.61 plants/m^2^, 18.75%). The lowest density (0.06 plants/m^2^) was recorded for *Pimpinella acuminata* (Table 4).

#### Altitudinal zone-IV (2900–3100 m)

In this altitudinal zone-IV, three tree species, nine shrub species and twenty two herb species with medicinal values were encountered (Table 2, Table 3 and Table 4). In the tree layer, the highest density (0.35 trees/100 m^2^) and frequency (25%) was found for *Viburnum cotnifolium* followed by *Abies pindrow*. The lowest density and frequency (0.10 trees/100 m^2^, 10%) was observed for *Taxus baccata* (Table 2). For shrub species, the highest density and frequency (2.73 plants/25 m^2^, 70%) was recorded for *Coriaria nepalensis* while the lowest density (0.35 plants/25 m^2^) was recorded for *Persicaria polystachya*. *Leycesteria formosa* and *Persicaria polystachya* had the lowest frequency (7.50%) (Table 3). Among the herb species, the highest density (1.15 plants/m^2^) and frequency (38.75%) was observed for *Rubus nepalensis* followed by *Ainsliaea apetra* (0.95 plants/m^2^, 31.25%). The lowest density and frequency (0.05 plants/m^2^, 2.50% respectively) was found for *Artemisia roxburghiana*, followed by *Bergenia ciliata* and *Impatiens scabrida* (Table 4).

#### Altitudinal zone-V (3350–3550 m)

Only two trees species with medicinal value were reported in the altitudinal zone-V. *Sorbus aucuparia* had the highest density and frequency (0.25 trees/100 m^2^, 25%), while *Abies pindrow* followed (0.10 trees/100 m^2^, 10%) (Table 2). Of the three shrub species encountered *Rosa sericea* was most common (1.23 plants/25 m^2^), followed by *Lonicera angustifolia* (0.38 plants/25 m^2^), and *Cotoneaster microphyllus* (0.23 plants/25 m^2^). The highest frequency (33.33%) was observed for both *Cotoneaster microphyllus* and *Lonicera angustifolia*, while *Rosa sericea* was much less frequent (26.53%) (Table 3). Among the thirty one herbs *Rumex nepalensis* (0.71 plants/m^2^, 27.50%), and *Ranunculus hirtellus* (0.38 plants/m^2^, 20%) had the highest density and frequency. The lowest density value (0.05 plants/m^2^) was found for *Maianthemum purpureum*, while *Bistorta amplexicaulis* was observed with lowest frequency (2.50%) (Table 4).

### Ethnomedicinal study of plant species

Of the total one hundred and fifty two species of ethnomedicinal plants complied for ethnomedicinal uses in the Himalayan region and Kedarnath Wildlife Sanctuary areas including 49 plant species of these were too reported from both the villages (Gundhaar and Ransi) of study area of Madhmeshwar, in KWLS. The scientific names, part used and ethnonomedicinal uses of these plants reported from the villages Gundhaar and Ransi is shown in Table 5. The reported 49 ethnomedicinal plants used to cure several ailments such as fever, cough, pain, wounds, cuts, insecticides, diarrhoea, dysentery, kidney problems, eye diseases, stop bleeding, abdomen pain, indigestion, antiseptic, healing foot cracks, mouth wash, blood diseases etc. The contribution of plant parts used by the inhabitants of Gundhaar and Ransi villages, was reported highest for roots (32%), followed by leaves (27%). Flowers, seeds and fruits contributed 8% for each and lowest contribution was reported for barks and resin of 3% and 1% respectively.

**Table 5:** Medicinal uses of plant species reported from the present study area

**Scientific name**	**Present study**	
	**Plant part used**	**Medicinal uses**
*Aconitium hetrophyllum* Wallich	Root	Fever and cough
*Aesculus indica* (Wall. ex Cambess.) Hook.f.	Seed	Rheumatic pain
*Anaphalis margaritaceae* (L.) Benth	Leaves	Wounds and cuts
*Anemone rivularis* Buch.-Ham. ex DC	Leaves	Wounds
*Artemisia japonica* Thunb.	Leaves	Insecticide
*Asparagus filicinus* Buch.-Ham. ex D. Don	Root	Diarrhoea and dysentery
*Barleria cristata* L.	Root	Wounds
*Bergenia ciliata* (Haw.) Sternb.	Root	Fever, kidney calculi, diarrhoea
*Blumea lanceolaria* (Roxb.) Druce	Leaves	Cuts
*Dicliptera bupleuroides* Nees	Leaves	Skin diseases, cough, wounds
*Elsholtzia strobilifera* Benth.	Whole plant	Wounds
*Eupatorium odenophorum* Spreng.	Leaves	Skin diseases
*Galium aparine* L.	Roots	Eye diseases and stop bleeding
*Geranium wallichianum* D. Don ex Sweet	Root	Dysentery and cold
*Girardiana diversifolia* (Link) Friis	Whole plant	Abdomen pain and indigestion
*Hippophae salicifolia* D.Don	Fruits	Dandruff
*Indigofera heterantha* Wall. ex Brandis	Leaves	Dysentery and cough
*Juglans regia* L.	Leaves	Insecticides
*Jurinea dolomiaea* Boiss.	Root	Incense
*Leycesteria formosa* Wallich	Leaves	Lice killing
*Morina longifolia* Wall. ex DC.	Root	Antiseptic, Burns, wounds
*Myrica esculenta* Buch.-Ham. ex D. Don	Leaves and fruits	Skin diseases and wounds
*Origanum vulgare* L.	Leaves	Tooth ache
*Pedicularis hoffmeisteri* Klotz.	Whole plant	Indigestion
*Phalaris minor* Retz.	Root	Wounds
*Picrorhiza kurrooa* Royle ex Benth.	Root	Stomach ache
*Pinus roxburghii* Sargent	Resin	Healing foot cracks
*Podophyllum hexandrum* Royle.	Root	Antiseptic, wounds
*Polygonatum verticillatum* (L.) All.	Root	Gastric problems
*Primula denticulata* Sm.	Root and flower	Lice killing
*Prinsepia utilis* Royle	Seed and roots	Stomach problems
*Pyrus pashia* Buch.-Ham. ex D. Don	Fruits	Digestive disorders
*Ranunculus hirtellus* Royle.	Whole plant	Wounds and cuts
*Reinwardtia indica* Dumort.	Flower juice	Mouth wash
*Rhododendron arboreum* Smith	Flower juice	Health tonic
*Roscoea alpina* Royle	Root	Urinary infections
*Rubia manjith* Roxb. ex Fleming	Flowers	Health tonic
*Rubus nepalensis* (Hook.f.) Kuntze	Root	Burns
*Sapindus mukorossi* Gaertn.	Fruit and seeds	Hair and antiseptic
*Sarcococca saligna* (D.Don) Muell.-Arg.	Leaves	Bone and muscle pains
*Silene edgeworthii* Bocquet.	Tender plant parts	Eye infections
*Swertia chirayita* (Roxb. ex Fleming) Karsten	Leaves	Fever and blood diseases
*Synotis alatus* (Wall. ex DC.) C. Jeffrey & Chen.	Whole plant	Fever
*Taxus baccata* L.	Bark	Breast infection
*Urena lobata* L.	Root	Muscle pains
*Urtica ardens* Link.	Leaf and seeds	Skin and hair diseases
*Urtica dioica* L.	leaves	Hair wash
*Veronica anagallis-aquatica* Linn.	Whole plant	Wounds and burns
*Zanthoxylum armatum* DC	Bark	Tooth ache

Ethnobotany explains the holistic relationships between plants and people [36]. Rapid global biodiversity loss is an issue of critical concern, with approximately 5000 species of animals and 25,00 species of plants currently listed as endangered, threatened, or at risk of overexploitation [37]. The Himalayan range is rich in endemic and medicinal plant diversity [38]. Uncontrolled developmental activities are causing a great loss to the biodiversity in the Indian Himalayan region, where medicinal plants in particular are declining at a very fast rate due to their over exploitation for trade [39], and it is believed that excessive anthropogenic activities are the main cause of decline in the population and availability of medicinal plants in the region [38,40]. There are many protected areas (PAs) across the Himalayan region but not a single PA has been specifically established to ensure the conservation of medicinal plants.

The plant species reported from the Madhmeshwar area of KWLS were one hundred and fifty two species having medicinally important value with one hundred twenty three genera belonging to sixty one families. In comparison [41] explored the Pindari area of Nanda Biosphere Reserve and reported 224 plant species with medicinal values. [42] recorded 701 species of medicinal plants of which 138 species were trees, 135 shrubs and 421 were herbs in various forest types of Uttarakhand. [43] presented a list of 41 medicinal plants with their medicinal uses and mode of application of Pauri Garhwal Himalaya. [44] reported 135 species having medicinal values from the Panwalikantha at an elevation of 3800 m. [45] reported a total of 335 medicinal plant species from the high altitude cold desert areas of Lahul-Spiti in Ladkh of which 45 were rare and endangered. [46] reported 228 species with medicinal and aromatic properties from Renuka Wildlife Sanctuary of Himalaya. Many of these medicinal plants are under of threat due to their heavy extraction [47]. A total of 1748 species having medicinal value have been reported from Indian Himalayan Region [15] contributing 90% of raw material for herbal industries in India and for export [48]. World trade figures suggest that India ranks next to China exporting raw material of medicinal plants [49].

The most commonly used parts of ethnomedicinal plants as collected through different literature survey were leaves (32%), roots (24%), whole plants or plant (13%), followed by fruits (9%) and seeds and flowers (6% each). This corroborates with [50] who also found that leaves were the most frequently used plant parts (48%) followed by stem bark (16%), roots and root bark (10%), while the fruits, whole plant, and aerial parts accounted for less than 10% for each. [19] reported that a single plant may be used for curing more than one ailment and observed that roots and root based preparations are the most used plant parts. [51] reported from Nepal that bark, flower, fruit, leaf, root, rhizome, tuber, seed, shoot, resin, and wood were used in this sequence.

In the study eighteen endangered plant species were found. [52] reported 37 species from Nanda Devi Biosphere Reserve as critically endangered, endangered, vulnerable and low risk near threatened using IUCN criteria. [15] reported that, as a result of over extraction 3.5% of the medicinal plants of the Indian Himalayan Regions (IHR) fall in different categories of threats.

In the study, the density and frequency for *Aconitium heterophyllum* was 0.33 plants/m^2^ and 18.75% while for *Jurinea dolomiaea* it was 0.08 plants/m^2^ and 3.75% respectively. *Picrorhiza kurrooa* was found with density of 0.23 ind/m^2^ having frequency of 12.50% while *Podophyllum hexandrum* with 0.19 plants/m^2^ density and 11.25% frequency. [53] reported 0.33 ind/m^2^ density of *Aconitium heterophyllum* in a part of Kedarnath Wildlife Sanctuary and [54] in Gori valley reported a total 0.465 ind/m^2^, and [55] found a density of 1.0 ind/m^2^ at Hari Ki Dun area to 2.57 ind/ m^2^ in Tungnath area of Garhwal Himalaya. [56] reported 2.721 ind/m^2^ and 86% values of density and frequency for *Jurinea dolomiaea* in alpine meadows of Kumaun Himalaya. [57] reported a density of 7 ind/m^2^ and frequency of 100% for *Jurinea dolomiaea* in rocky areas in alpine area of Chhota Bhangal in Himachal Pradesh. Working on the population density of *Picrorhiza kurrooa*, [54] reported a density of 3.89 ind/m^2^ from upper Gori valley and 4.5 ind/m^2^ in the valley of Flowers National Park, while [53] reported density values of 3.36 ind/m^2^ in Kedarnath Wildlife Sanctuary. [47] reported 2 ind/m^2^ density of *Podophyllum hexandrum* in Pin Valley National Park, while [58] reported 21.8 to 94.73 ind/m^2^ density and [54] reported 0.193 ind/m^2^ in Gori valley, with a density of 0.98 ind/m^2^ in the Valley of Flowers National Park and 0.72 ind/m^2^ in Kedarnath Wildlife Sanctuary. In contrast [59] found only density values of 0.012 ind/m^2^ and a frequency value of 18.70% in its natural habitats in Kashmir Himalaya.

The study indicates that in-depth phytochemical and pharmacological investigations would be of interest for some plants with unique or lesser known medicinal applications. The conservation of plant biodiversity in the Indian Himalayan region has become a major concern and more detailed studies on population structure and regeneration rates are needed to plan conservation measures. The traditional knowledge of plant species as medicine is vanishing rapidly, and traditional health care systems are disappearing, and the oral transmittion of knowledge is clearly decreasing. Therefore, the knowledge of indigenous uses of native plants needs to be studied before it gets extinct [60].

## Conclusions

Considering the ecological importance and population status of important ethnomedicinal species, we recommend the preparation of micro-plans for each important medicinal species, including data on best harvesting practice and quantity to be harvested. Most of this data is unknown for most medicinal plants. Propagation of plants using tissue culture techniques and conventional methods to allow for their transplantation into natural habitats and niche areas of the species will be an important step towards their conservation. Additional ecological studies, including population assessments using standard ecological methods are needed to effectively plan the conservation and management for threatened, rare and endangered species. The development of agro-production techniques for certain species of Garhwal Himalaya can help to meet the requirement of raw material for commercial use and reduce the pressure on the existing populations in natural habitats.

## Competing interests

The authors declare that they have no competing interests.

## Authors’ contributions

JAB and MK complied the collected field data, analysed and draft the manuscript, RWB revised the manuscript added the valuable suggestions for manuscript improvement. All authors read and approved the final manuscript.
